# A Statistical Approach to Assess the Robustness of Radiomics Features in the Discrimination of Mammographic Lesions

**DOI:** 10.3390/jpm13071104

**Published:** 2023-07-07

**Authors:** Alfonso Maria Ponsiglione, Francesca Angelone, Francesco Amato, Mario Sansone

**Affiliations:** Department of Information Technology and Electrical Engineering, University of Naples Federico II, 80125 Naples, Italy; francesca.angelone@unina.it (F.A.); framato@unina.it (F.A.)

**Keywords:** radiomics, mammography, breast lesions, statistical analysis, robustness score

## Abstract

Despite mammography (MG) being among the most widespread techniques in breast cancer screening, tumour detection and classification remain challenging tasks due to the high morphological variability of the lesions. The extraction of radiomics features has proved to be a promising approach in MG. However, radiomics features can suffer from dependency on factors such as acquisition protocol, segmentation accuracy, feature extraction and engineering methods, which prevent the implementation of robust and clinically reliable radiomics workflow in MG. In this study, the variability and robustness of radiomics features is investigated as a function of lesion segmentation in MG images from a public database. A statistical analysis is carried out to assess feature variability and a radiomics robustness score is introduced based on the significance of the statistical tests performed. The obtained results indicate that variability is observable not only as a function of the abnormality type (calcification and masses), but also among feature categories (first-order and second-order), image view (craniocaudal and medial lateral oblique), and the type of lesions (benign and malignant). Furthermore, through the proposed approach, it is possible to identify those radiomics characteristics with a higher discriminative power between benign and malignant lesions and a lower dependency on segmentation, thus suggesting the most appropriate choice of robust features to be used as inputs to automated classification algorithms.

## 1. Introduction

Breast cancer is a multifactorial disease, characterized by the uncontrolled multiplication of mammary gland cells [1]. The major risk factors associated with breast cancer can be classified into: (i) nonmodifiable risk factors (such as age, gender, genetic factors, a family history of breast cancer, previous breast cancer, and/or proliferative breast disease); (ii) modifiable risk factors (such as reproductive factors, radiation exposure, hormone replacement therapy, alcohol, and a high-fat diet); (iii) environmental factors (such as exposure to organochlorines, electromagnetic fields, and smoke) [2,3,4]. Although there is not an absolute correlation between the disease and the above-mentioned factors, they are assessed in the anamnesis phase and can influence the diagnosis [1]. According to national reports [5], breast cancer ranks first in terms of mortality level among the cancer pathologies affecting the female population worldwide, particularly in the 35–55 age group, with 6–7% of cases being metastatic at the time of the diagnosis [5]. Since the chances of recovery strongly depend on the tumour grade at the time it is diagnosed, early detection is of fundamental importance. Indeed, advanced-grade breast cancers could require invasive interventions followed by heavy radiotherapy and chemotherapy treatments [6,7,8]. On the other hand, early-stage tumours can often require less invasive therapeutic approaches, with increased success rates. Therefore, screening protocols and campaigns can serve as helpful approaches to foster the early diagnosis of breast cancer [9,10,11].

In this regard, mammography (MG) is among the most widespread techniques to perform screening exams for the detection of small nodules, potentially dangerous calcifications, and other types of suspicious lesions [12,13]. The screening exam consists of an X-ray scan of each breast in two projections (or views), namely the craniocaudal (CC) and the medial lateral oblique (MLO) projection, to improve anomaly visualization, thus obtaining four images per patient. Based on the signs/symptoms or the patient’s history, a further mammogram may be required. Therefore, MG results in an X-ray low-dose greyscale digital image representing the structure of the breast [14].

Despite its ability to detect 85–90% of breast cancers early, before a medical examination [15], several misdiagnosis cases and errors are still reported and can be attributable to the following main reasons: (i) the nature of the abnormality, which exhibits a high variability in its morphological characteristics; (ii) the similarity between potentially malignant masses and surrounding healthy tissues, especially occurring in young women with dense fibrous breast tissue, which appears opaque in MG. In order to overcome these issues, computer-aided detection and diagnosis (CAD) systems have been introduced to assist radiologists in the interpretation of the medical images and in segmenting, extracting, processing, and classifying imaging features within suspicious regions of interest (ROIs) to determine the phenotypic characteristics of the abnormalities, thus helping to differentiate malignant abnormalities from benign ones [16]. Indeed, by means of radiomics, which consists of an advanced extraction and analysis of textural features from medical images, quantitative parameters and measures of tumour heterogeneity can be obtained, thus supporting the interpretation of the radiology findings [17].

Studies from the literature have shown that a double reading from two independent radiologists can improve the diagnosis of breast lesions [18,19,20,21]; however, a double check has a high operational cost in terms of resource utilization and time, thus preventing its application in broader healthcare settings. In this sense, CAD systems, along with reproducible and standardized radiomics workflows, could offer the opportunity to obtain a “second opinion” from an automated tool, thus improving the diagnostic accuracy with less operational cost and within a reduced timeframe [22,23]. The sensitivity of such automated tools, i.e., the ability to find and properly characterize suspicious areas, is, therefore, of fundamental importance to increase the reliability and clinical translation of CAD systems and radiomics workflows in most radiology applications [24,25,26,27,28,29,30,31,32], which can be then combined with artificial intelligence (AI) approaches, such as machine learning (ML) and deep learning (DL) techniques, to further boost the automated diagnostic performance [33,34,35,36,37,38,39,40,41].

To ensure the reliability, robustness, and reproducibility of CAD systems to process and classify medical images, and to facilitate and support classification tasks, an accurate and appropriate choice of radiomics features is of utmost importance, by selecting those that better describe and reflect the tissue properties and lesion characteristics. In this context, the main objective of the present work is to examine radiomics features’ robustness and variability depending on the segmented ROI of breast masses and calcification from a public database of MG images. An automated technique is adopted to generate artificial ROIs, namely underestimated and overestimated regions, starting from the original ROI associated with the MG image. A statistical approach is carried out to assess the variability of radiomics features across the original and the artificially generated ROI, and a robustness score is provided.

## 2. Methods

### 2.1. Dataset

The study is carried out on a sample of 622 digitized mammograms from the public database “Curated Breast Imaging Subset of Digital Database for Screening Mammography” (CBIS-DDSM) [42,43], provided by The Cancer Imaging Archive (TCIA) [44]. The images are DICOM images obtained through a lossless decompression of the original Digital Database for Screening Mammography (DDSM) images, distributed in a Lossless Joint Photographic Experts Group (LJPEG) format. The database includes instances with certified pathology data for normal, benign, and cancerous conditions, and is designed to serve as a tool for testing and improving decision support and CAD systems. For the purpose of this work, only breast projections containing abnormalities were considered. The type of anomaly between calcifications and masses, or both, is specified in the metadata associated with the database, and the classification of the abnormalities between malignant and benign is also indicated. Out of the 622 images considered in this study, 268 concerned abnormalities classified as calcifications (146 MLO views and 122 CC views), and 354 concerned abnormalities classified as masses (188 MLO views and 166 CC views).

### 2.2. Methodological Workflow

**Figure 1:**
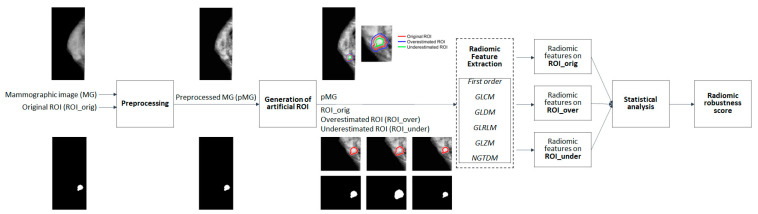
Graphical representation of the study workflow with main methodological steps. (FO: first order; GLCM: grey-level co-occurrence matrix; GLDM: grey-level dependence matrix; GLRLM: grey-level run length matrix; GLSZM: grey-level size zone matrix; and NGTDM: neighbourhood grey tone difference matrix.)

The main steps of the adopted methodological workflow are shown in Figure 1.

A preprocessing phase was followed by the generation of two artificial ROI based on the original segmentation provided with the input image. Both overestimated and underestimated segmentations were produced and given as inputs to a radiomics feature extraction process based on PyRadiomics [45]. Six groups of features were extracted from each segmentation and a statistical analysis phase was then performed to assess the variability in the features across the different ROIs and to derive a robustness score based on the results of the statistical analysis.

In the following subsections, a detailed explanation of each phase of the methodological workflow displayed in Figure 1 is provided.

### 2.3. Preprocessing

The examination of mammograms for breast cancer screening and diagnosis requires a preprocessing step, which has been shown to be of utmost importance to reduce the incidence of false-positive results [46]. In this work, image preprocessing was carried out by means of contrast limited adaptive histogram equalization (CLAHE), as presented and suggested by Tripathya and Swarnkarb in [46] and by Zuiderveld Karel [47].

**Figure 2:**
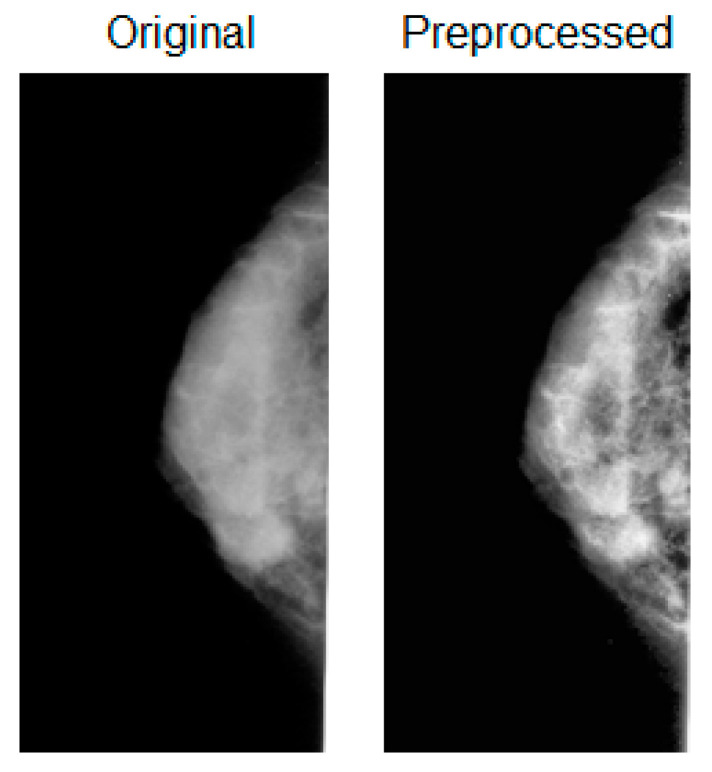
Illustration of original and preprocessed MG images in CC view.

Figure 2 shows a comparison between the original and preprocessed images.

The adopted preprocessing algorithm enhances the contrast in rectangular discrete sections in which the original image is divided and implements a bilinear interpolation to join the adjacent sections. The algorithm is implemented in MatLab (R2021b, The Mathworks, Inc., Natick, MA, USA). Subsequently, the preprocessed image, along with the associated ROI binary mask representing the original segmentation, is passed to an automated algorithm for the generation of synthetic ROI binary masks based on the original one, as detailed in the following subsection.

### 2.4. Generation of Artificial ROI

An automated algorithm for the generation of artificial ROI binary masks based on the overestimation and underestimation of the original segmentation was proposed and carried out in MatLab (R2021b, The Mathworks, Inc., Natick, MA, USA). The algorithm takes the original image and associated original segmentation as inputs and automatically applies both a binary morphological dilation and an erosion operation on the input ROI, taking advantage of the decomposition of a structuring element as described in [48,49], in order to generate both an overestimated and an underestimated ROI binary mask. An octagonal structuring element is chosen for both dilation and erosion operations. The extent or the scale factor of the morphological operation, i.e., the distance between the structuring element origin and its sides, is proportional to the original ROI main dimension, taken as the length (expressed in pixels) of the major axis of the ellipse that has the same normalized second central moments as the original ROI. The scale factor of the dilation and erosion was chosen to be proportional to the original ROI main dimension. The constant of proportionality between the scale factor of the morphological operation and the major axis of the original ROI was empirically set to 0.06 (i.e., the original ROI was dilated/eroded to an extent equal to 6% of its major axis). This allowed us to achieve perceptible changes with respect to the original ROI while avoiding excessive dilation or erosion.

**Figure 3:**
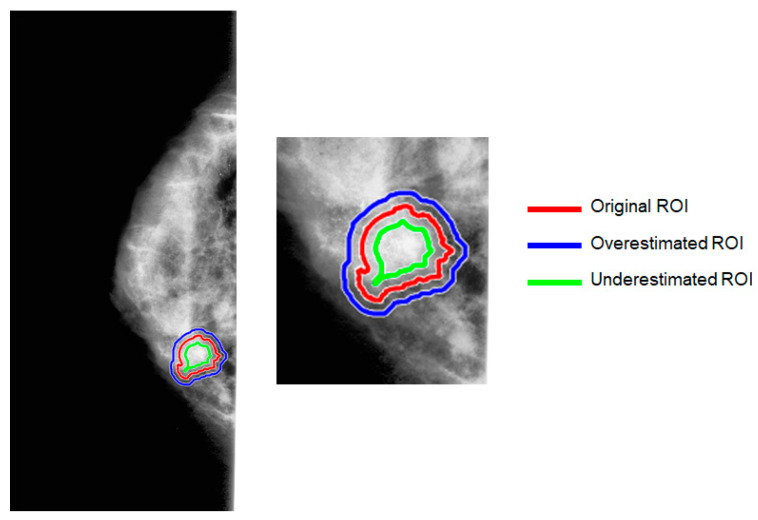
Illustration of original and artificially generated ROI contours. Artificial ROI represents both overestimated and underestimated abnormality segmentations with respect to the original ROI.

Figure 3 provides a graphical representation of the contours of the artificial segmentations compared with the original one.

**Figure 4:**
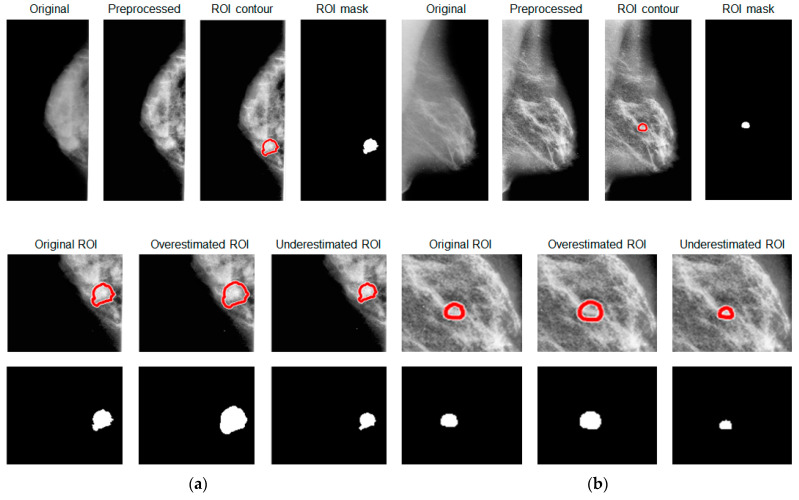
Visual comparison of original and artificially generated ROI masks and contours on (**a**) MLO and (**b**) CC image view, respectively.

In Figure 4, original and artificially generated ROI masks and contours are reported for mammograms in both MLO and CC views.

Both original, overestimated, and underestimated segmentations were given as inputs to a radiomics feature extraction process based on PyRadiomics [45], as detailed in the following subsection.

### 2.5. Radiomics Feature Extraction

Once artificial ROIs are generated, a feature extraction process using PyRadiomics version 3.0.1 was carried out. PyRadiomics is an open-source Python library [45] for the extraction of radiomics data from medical images in compliance with the Imaging Biomarker Standardisation Initiative (IBSI). Values of different statistical and textural features are calculated on the preprocessed MG image within each ROI, both corresponding to the original and artificial segmentations. Radiomics features are usually divided according to the characteristic to which they refer: those related to the morphology of the lesion and those related to the texture. In the former case, the size and shape of the lesion are studied, while the latter, being linked to the image texture, are based on the histogram of the grey levels and the corresponding matrix of the pixels forming the image. Here, six groups of radiomics features are computed. These consist of first-order (FO) features, and the following second-order features: grey-level co-occurrence matrix (GLCM), grey-level dependence matrix (GLDM), grey-level run length matrix (GLRLM), grey-level size zone matrix (GLSZM), and neighbourhood grey tone difference matrix (NGTDM).

The FO operators are statistical operators based on the image brightness histogram and, thus, describe the distribution of values related to individual pixels. Although not providing any information on a morphological level, they allow obtaining a first estimate of the brightness and homogeneity of the image. Indeed, based on the histogram, statistical parameters can be considered to estimate the radiomics features, such as the mean (representing the average intensity that the grey levels can assume), the variance (representing a measure of the spread of the distribution with respect to the mean, in other words, an indirect measure of the image contrast), the skewness (i.e., the symmetry of a distribution with respect to the mean), the kurtosis (representing the peak shape of the ROI values’ distribution, with higher kurtosis), and the entropy (representing the randomness in the pixel values). Unlike the FO features, the second-order ones were calculated using second-order statistics and provided an evaluation of the spatial dependence between the values associated with individual pixels. The descriptors were calculated on the co-occurrence matrix, or GLCM, which was calculated starting from an image I of dimensions *n* × *m* by determining how many times a pixel with intensity *i* was spatially related with a pixel of value *j*. Each element (*i*, *j*) in the resulting matrix was equal to the sum of the occurrences in which the *i-th* pixel was related to *j-th* pixel of the image. Concerning the GLDM, it represents the grey-level dependencies in an image, i.e., the number of pixels connected to and dependent on the centre pixel of the image within a given distance, δ. As far as the GLRLM is concerned, it records the occurrence of all various combinations of grey-level values and grey-level runs in an ROI for a given direction, typically along four principal directions (i.e., horizontal, antidiagonal, vertical, and diagonal). Regarding the GLSZM, it is a matrix representation of a 2D histogram of the total number of zones of size *j* and of intensity value *i*. Finally, the NGTDM stores the sum of absolute differences for a given grey-level *i* by quantifying the difference between the reference grey value and the average grey value of its neighbours within a given distance δ.

The feature values obtained for all the groups were collected in a CSV file before undergoing a statistical analysis, as discussed in the following section.

### 2.6. Statistical Analysis and Radiomics Robustness Score

The data obtained from the feature extractor were then collected in an CSV file, with the first columns reporting the metadata associated with each processed record and patient, and the subsequent columns including the values for each calculated radiomics feature. A statistical analysis was carried out on both abnormality type (masses or calcifications) and pathology type (benign or malignant) separately. The data were grouped according to the following segmentation classes: original ROI, overestimated ROI, and underestimated ROI, in order to assess the robustness of the radiomics features across the three examined ROI. A first analysis was performed on the whole sample, i.e., considering both MLO and CC views. Then, the same analysis was reiterated, taking into account each image view (MLO and CC) separately in order to investigate the robustness and variability of radiomics features at each specific projection.

A Shapiro–Wilk test (α = 0.05) was carried out to assess the normality of the distribution of the radiomics features across the groups to be compared. Based on the results of the normality checks, a parametric (or nonparametric) test, namely ANOVA (or Kruskal–Wallis), was performed (α = 0.05) to compare the radiomics features distributions across the three segmentation classes according to pathology type (benign and malignant), abnormality type (masses and calcifications), and view type (MLO or CC). Based on the *p*-value of the statistical test, an “overall robustness score”, ranging from 0 to 1, was defined as equal to the significance of the test. In this way, the closer to 1 the overall score is, the less statistically significant the test (high *p*-value) is, thereby reflecting a higher robustness in the examined radiomics feature, whose distribution did not show significant variations across the ROI segmentations. For example, if the Kruskal–Wallis statistical test carried out on a specific radiomics feature returned a *p*-value equal to 0.02 (i.e., less than the significance level α), it could be concluded that a statistically significant difference existed among the median values assumed by the examined radiomics features in the three segmentations (original vs. underestimated vs. overestimated). As a consequence, we could hypothesize that the examined radiomics feature varied significantly across the different segmentations of the same ROI and, therefore, it lacked robustness. With the aim of quantifying the robustness, the “overall robustness score” attributed to the examined radiomics feature was chosen to be equal to 0.02, i.e., equal to the probability value (*p*-value) of the employed statistical test. Conversely, if the *p*-value was equal to 0.80, it could be concluded that the median values of the examined radiomics feature did not significantly differ across the three explored segmentations, leading us to the hypothesis that the examined feature is robust, with an “overall robustness score” of 0.80.

Since the above-mentioned test indicated that at least one difference existed among the classes, but did not identify where the stochastic difference occurred, a parametric (or nonparametric) test, namely Student’s t-test (or the Mann–Whitney U-test), was performed to make a pairwise comparison of the radiomics feature distributions between the original ROI and the overestimated ROI as well as between the original ROI and the underestimated ROI, in order to assess the feature robustness at both dilation and erosion transformation of the segmented area. Like the overall robustness score, a “segmentation-specific robustness score” (hereafter referred to as the “robustness score”) is defined as equal to the *p*-value of the test. For example, as for the overall robustness score, if the *p*-value of 0.02 (i.e., less than the significance level α) is obtained for the Mann–Whitney U-test to compare the median values of certain radiomics features between the original ROI and the overestimated ROI, then we can conclude that a statistically significant difference exists between the feature median values in the two classes (original vs. overestimated ROI). Therefore, we could hypothesize that the examined radiomics feature lacks robustness and, thereby, we could attribute a low robustness score (0.02) equal to the *p*-value of the test. Conversely, if a *p*-value of 0.80 is obtained with the same test, it can be concluded that the median values of the feature measured on the original ROI do not significantly vary on the overestimated ROI, leading us to the hypothesis that the examined feature is robust against ROI overestimation, with an “overall robustness score” of 0.80. The definition of a more specific score is necessary to achieve a better and wiser assessment of the robustness by considering the effects of ROI dilation and erosion separately.

Finally, boxplots were used to visually represent the obtained results in terms of robustness scores. In particular, the variability of the scores calculated for the six different feature classes (FO, GLCM, GLDM, GLRLM, GLSZM, and NGTDM) was shown through boxplots by grouping the data according to the abnormality type (calcifications or masses), pathology type (benign or malignant), and view type (MLO or CC). Boxplots were also used to carry out a visual analysis on a representative set of radiomics features to illustrate the variability at different robustness levels (high vs. low, qualitatively defined according to the robustness score) and at different levels of discriminative power (high vs. low, qualitatively defined according to the ability to discriminate between benign and malignant lesions).

All the feature elaborations and statistical tests were carried out in R language within the RStudio environment.

## 3. Results

**Figure 5:**
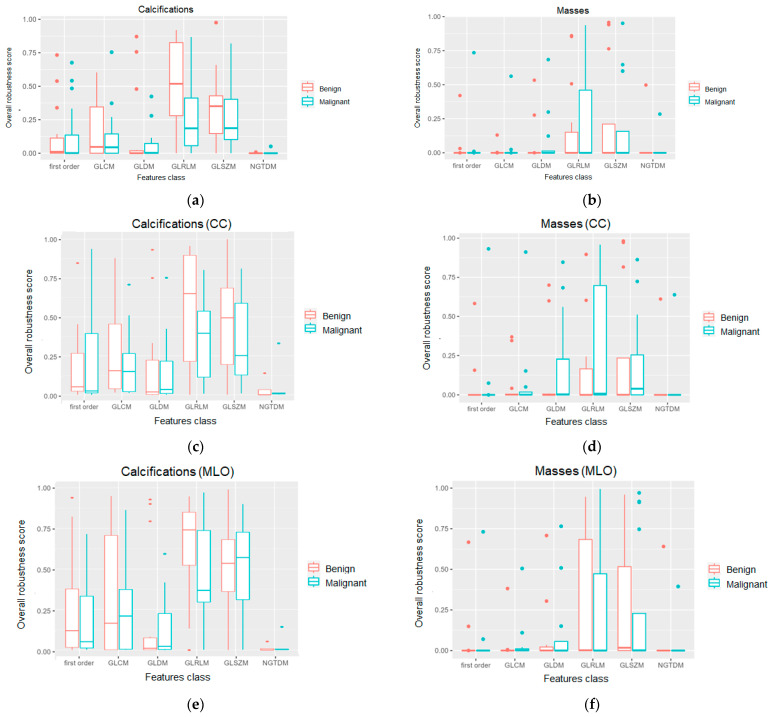
Distribution of the overall robustness scores in the different radiomics feature classes calculated for: (**a**) calcifications, both MLO and CC view considered together; (**b**) masses, both MLO and CC view considered together; (**c**) calcifications in CC view only; (**d**) masses in CC view only; (**e**) calcifications in MLO view only; (**f**) masses in MLO view only. (FO: first order; GLCM: grey-level co-occurrence matrix; GLDM: grey-level dependence matrix; GLRLM: grey-level run length matrix; GLSZM: grey-level size zone matrix; and NGTDM: neighbourhood grey tone difference matrix).

In Figure 5, the distributions of the overall robustness scores calculated for both masses and calcifications are represented. The results are differentiated according to the pathology type (benign or malignant) and according to the image view (MLO and CC).

It can be observed that the radiomics features calculated for calcifications showed higher overall robustness scores, especially for the FO, GLCM, GLRLM, and GLSZM categories (Figure 5a), compared to the corresponding ones computed for masses (Figure 5b). This is far more evident when comparing the scores obtained for CC and MLO views in the calcification subset (Figure 5c,e) with those in the mass subset (Figure 5d,f).

**Figure 6:**
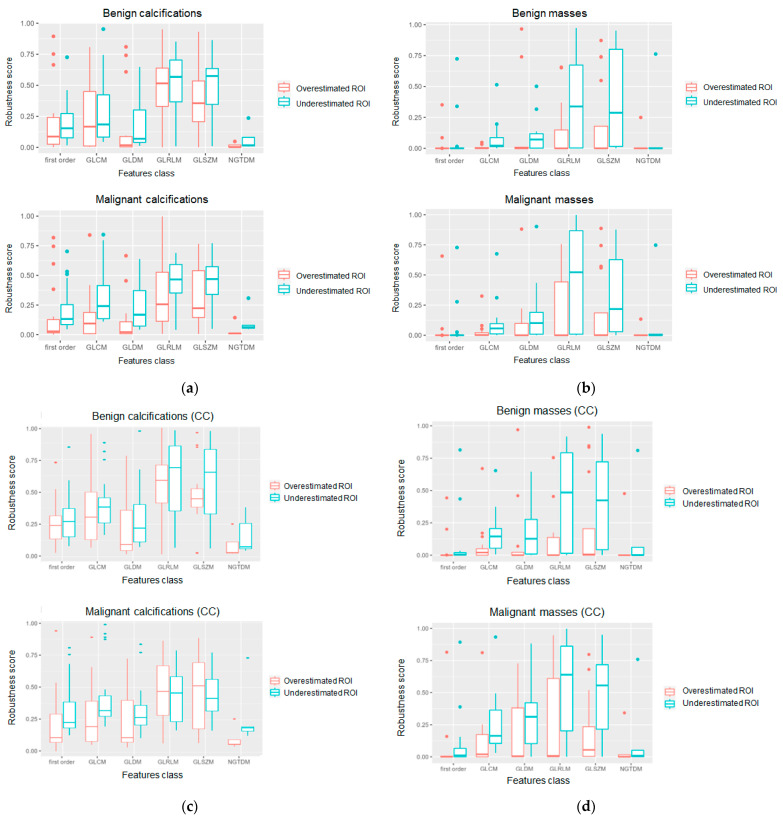

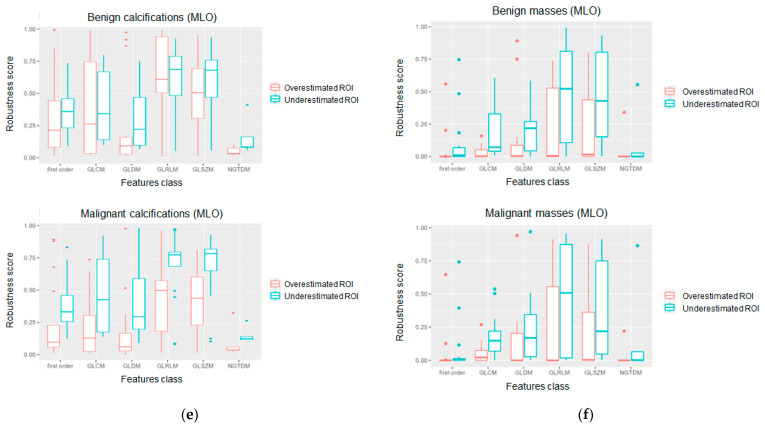
Distribution of the robustness scores in the different radiomics feature classes calculated for: (**a**) benign and malignant calcifications, both MLO and CC view considered together; (**b**) benign and malignant masses, both MLO and CC view considered together; (**c**) benign and malignant calcifications in CC view only; (**d**) benign and malignant masses in CC view only; (**e**) benign and malignant calcifications in MLO view only; (**f**) benign and malignant masses in MLO view only. (FO: first order; GLCM: grey-level Co-Occurrence matrix; GLDM: grey-level dependence matrix; GLRLM: grey-level run length matrix; GLSZM: grey-level size zone matrix; NGTDM: neighbourhood grey tone difference matrix).

Figure 6 displays the distributions of the robustness scores in the different radiomics feature classes calculated for masses and calcifications. From this figure, it is possible to investigate the segmentation-specific robustness by separately and independently observing the behaviour of the robustness score after ROI dilation (overestimated ROI) and ROI erosion (underestimated ROI). The results are differentiated according to the pathology type (benign or malignant) and according to the image view (MLO or CC).

It can be observed that the radiomics features calculated on the underestimated ROI in for calcifications (Figure 6a) and masses (Figure 6b) were far more robust than the respective features calculated on the overestimated ROI, thus confirming a nondesirable impact of the dilation operation on the consistency of the radiomics feature distributions. This consideration appears to be applicable to all feature categories in the calcification subset (Figure 6a), while only to GLCM, GLDM, GLRLM, and GLSZM classes in the mass subset (Figure 6b). Similar behaviour was also observable when grouping the data according to image view type (MLO and CC), with higher robustness scores in the calcification dataset (Figure 6c,e) compared to the corresponding ones computed for masses (Figure 6d,f).

**Figure 7:**
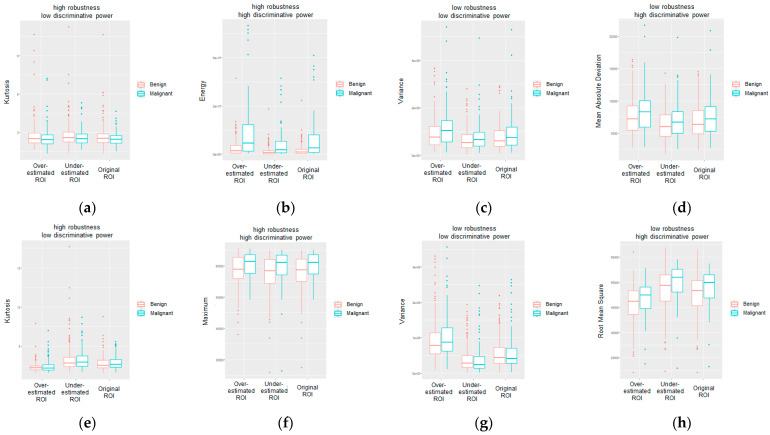
Representative examples of FO radiomics features with different robustness levels (high or low) and different discriminative power (high or low) for: (**a**–**d**) calcifications; (**e**–**h**) masses.

A binary qualitative robustness level (low or high) is defined by ranking the calculated radiomics features based on their robustness score. Similarly, a binary qualitative discriminative power level (low or high) is defined through a visual observation of the feature values’ distribution across benign and malignant abnormalities. In Figure 7, some representative examples of radiomics features in the FO category with different robustness levels and different discriminative power are shown for both calcifications and masses.

Among the FO radiomics features, the kurtosis showed to be the one with the highest robustness score in both the calcification (Figure 7a) and mass (Figure 7e) subsets. Indeed, especially for calcification (Figure 7a), it can be observed that the distributions were consistent across the segmentations for both benign and malignant groups. Despite its robustness, the kurtosis showed a rather low discriminative power in distinguishing between benign and malignant abnormalities, as can be visually deduced from the boxplots in Figure 7a,e, with both showing almost overlapping median values between the benign and malignant groups in each segmentation. Similarly, the variance showed to be the one with the lowest discriminative power score in both the calcification (Figure 7c) and mass (Figure 7g) subsets. However, differently from the kurtosis, it also showed low robustness as the distribution values showed significant changes across the different segmentations in both benign and malignant cases. Moreover, further differences can be observed between the calcification and mass subsets. Indeed, the features showing both high robustness and high discriminative power were the energy in the calcification dataset (Figure 7b) and the maximum in the mass dataset (Figure 7f), while the features showing both low robustness and high discriminative power were the mean absolute deviation in the calcification dataset (Figure 7d) and the root mean square in the mass dataset (Figure 7h).

## 4. Discussion

Breast cancer accounts for the highest mortality rate among cancer pathologies affecting female population globally. In recent years, the mortality rate has decreased thanks to early diagnosis, especially through mammography (MG) screening campaigns for the detection of tumour lesions in the early stages to avoid invasive interventions and unnecessary therapies. However, despite MG being the most widespread technique to perform breast cancer screening, lesion detection and classification between benign and malignant tissues remains a complicated issue due to the great variability in the shape and size of lesions and their similarity to healthy tissues. The automated extraction of morphological, statistical, and textural features of medical images within a radiomics workflow proved to be helpful in aiding the early diagnosis of diseases.

Although several studies have investigated the repeatability of radiomics features, most of them focused on the following: a comparison across different manufacturers [50,51] and different image storage formats (raw vs. processed) [52] in full-field digital mammography (FFDM); a comparison between various AI-based feature extraction and segmentation approaches, e.g., radiomics and DL in magnetic resonance imaging (MRI) [53], manual and DL segmentation in in MRI [54,55], and computed tomography (CT) [56]; and the study of the impact of both clinical and radiomics features in contrast-enhanced spectral mammography (CESM) [57,58], in MG [59], and in FFDM [60]. Despite these valuable research efforts, and although the IBSI initiative addresses the issue of the reproducibility of radiomics characteristics, it is still difficult to interpret radiomics signatures and most studies have concentrated on repeatability using various imaging devices or reconstruction methods but employing one segmentation, which might not be accessible in clinical practice. Instead, from a clinical perspective, it is of utmost importance to assess the robustness of radiomics features regarding segmentation variability in order to identify those features to be included in the clinical evaluation.

In this study, the variability and robustness of radiomics features was investigated as a function of the lesion segmentation in MG images from a public database. A statistical analysis was carried out to assess feature variability and a radiomics robustness score was introduced based on the significance of the statistical tests performed. The major contributions of this work are three-fold: (i) the proposal of an approach to investigate and evaluate the variability in radiomics features as a function of the ROI size; (ii) the quantification of an overall robustness score based on the *p*-value of the statistical analysis of the radiomics features; (iii) the investigation of robustness across different radiomics categories, namely FO, GLCM, GLDM, GLRLM, GLSZM, and NGTDM.

As can be observed from the obtained results (Figure 5 and Figure 6), variability was observable not only as a function of the abnormality type (calcification and masses), but also among the feature categories (first-order and second-order), image view (craniocaudal and medial lateral oblique), and pathology (benign and malignant). The representative case of a FO radiomics feature (Figure 7) highlights that the energy was able to provide both an adequate robustness level and a good degree of discrimination between benign and malignant calcifications. The energy reflects the magnitude of pixel values in an image. A larger value, which is observed in malignant calcifications, could imply a higher intensity in the abnormality region. Similarly, the maximum was identified among the most robust and with higher discriminative power among the FO radiomics features calculated on breast masses. As in the case of the energy, the maximum reflects the intensity of the lesion area, and it showed larger values in malignant abnormalities compared to benign ones. The wise selection of most robust and predictive features along with their biological interpretation could serve as a basis to build resilient radiomics workflows in medical imaging and could provide novel insights in the definition of reliable indicators and biomarkers to detect malignancies and support in-depth analysis and diagnosis.

As far as the limitations of the present study are concerned, it is worth underlining that the statistical analysis carried out was univariate, as each variable was analysed individually without considering the interplay among the different extracted features. In future works, in order to improve the evidence provided by the present study, a multivariate analysis will be carried out by considering all the radiomics features simultaneously and performing a robustness assessment based on the obtained results. Furthermore, as the size of the dataset adopted in this study was limited to images from a public database referring to the same institution and image acquisition protocol, further studies could be focused on assessing the robustness at a multicentre level, including MG images acquired with different protocols and instruments, potentially providing additional insights into the variability and robustness of the radiomics features. Last but not least, future studies will also address the robustness of ML models across different segmentations by exploiting the workflow proposed here.

## 5. Conclusions

Radiomics is a promising method for identifying image-based indicators to support personalized diagnosis and treatment. The aim of this work was to investigate the robustness of radiomics features in MG as a function of the lesion segmentation to provide an automatic tool for designing a resilient and robust radiomics workflow based on the selection of segmentation-independent features with higher discriminative power to be used in abnormality characterization and classification tasks. In order to investigate and understand which radiomics characteristics can be considered most robust in MG, an algorithm was implemented to generate artificial segmentations based on an originally defined ROI, statistical analysis was carried out to assess the feature variability across different abnormality segmentations, and a radiomics robustness score was introduced based on the significance of the statistical tests performed. The results show that variability was observable not only as a function of the abnormality type (calcification and masses) but also among feature categories (first-order and second-order), image view (craniocaudal and medial lateral oblique), and pathology (benign and malignant). In particular, radiomics features were shown to be more robust while classifying breast calcifications rather than breast masses (as shown in Figure 5a,b and Figure 6a,b). Considering craniocaudal (CC) and mediolateral oblique (MLO) views separately, this difference between feature robustness across calcification and masses was even more evident (as shown in Figure 5c–f and Figure 6c–f). In order to investigate the predictive power of the features and their relative robustness, a representative example of the results obtained for the radiomics features of the first order is provided in Figure 7 of the manuscript. Indeed, in the case of breast calcifications, Figure 7a–d show how the energy turned out to be the most robust radiomics feature and the one with the highest discriminative power between benign and malignant lesions; conversely, in the case of breast masses, Figure 7e–h show how the maximum turned out to be the most robust radiomics feature and the one with the highest discriminative power between benign and malignant lesions. These results show that a preliminary selection of segmentation-independent and predictive features in MG images can be achieved and could suggest links between either statistical or textural image patterns and the biological/anatomical characteristics of the abnormalities, thus building a route for the definition of reliable descriptors to be adopted in CAD systems and to be used to test automated classification algorithms.

## Data Availability

Not applicable.
